# Supplementary material for: Rapid global ocean-atmosphere response to Southern Ocean freshening during the last glacial
Source: Nat Commun. 2017 Sep 12;8:520. doi: 10.1038/s41467-017-00577-6 (PMC5595922; doi:10.1038/s41467-017-00577-6)
Supplement: Supplementary file 1 — Supplementary Information [file 41467_2017_577_MOESM1_ESM.pdf]

Description of Supplementary Files

File Name: Supplementary Information
Description: Supplementary Figures, Supplementary Tables and Supplementary References

File Name: Supplementary Data 1
Description: WAIS Divide Ice core alternative age scale $WD2014_{sync}$. The WD2014 chronology is based on an annual layer count down to a depth of 2850 m. This alternative age scale uses interpolar methane synchronization to a linearly scaled version of the Greenland Ice Core Chronology (GICC'05) for the 2700-2850 m depth interval instead. This means the chronology is fully synchronized to Greenland for GI-3 to GI-18. This $WD2014_{sync}$ chronology is based on a linear scaling of GICC05; the stretching factor of 1.0063 is chosen to bring the Greenland time scale in better average agreement with absolute U/Th dated events in the Hulu speleothem record. All stated uncertainties are 2 sigma values. The total uncertainty stated is the relative uncertainty in the synchronization, and not the absolute age uncertainty.

File Name: Supplementary Software 1
Description: OxCal code for modelling radiocarbon ages.

File Name: Peer Review File

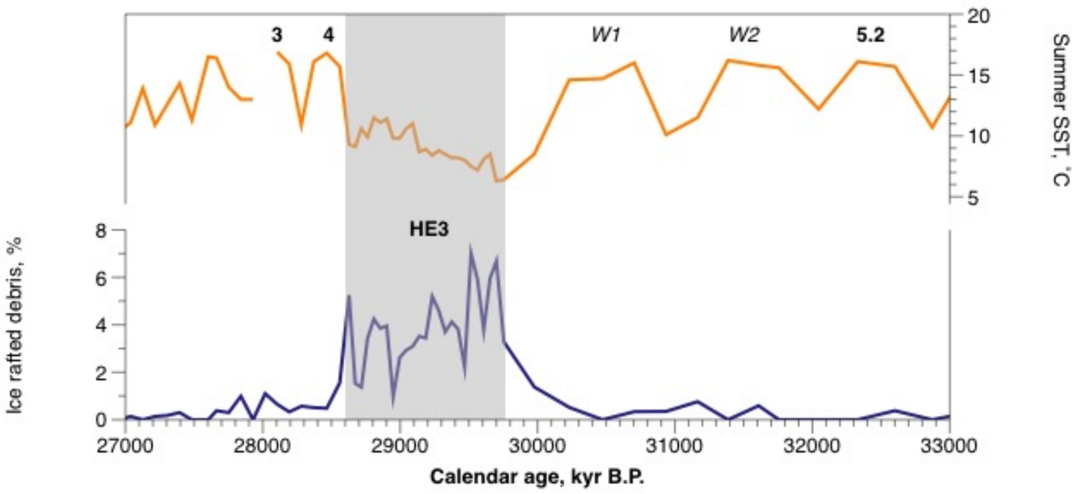

Supplementary Figure 1: North Atlantic record of climate and environmental changes between 27 and 33 kyr BP. North Atlantic (Iberian) ice rafted debris Heinrich event 3 (HE3; blue line) relative to reconstructed summer sea surface temperatures (orange line) in marine core MD95-2040 (ref. [1]). Note, warming events 1 and 2 (*W1* and *W2*) that fall between GI-4 and 5.2.

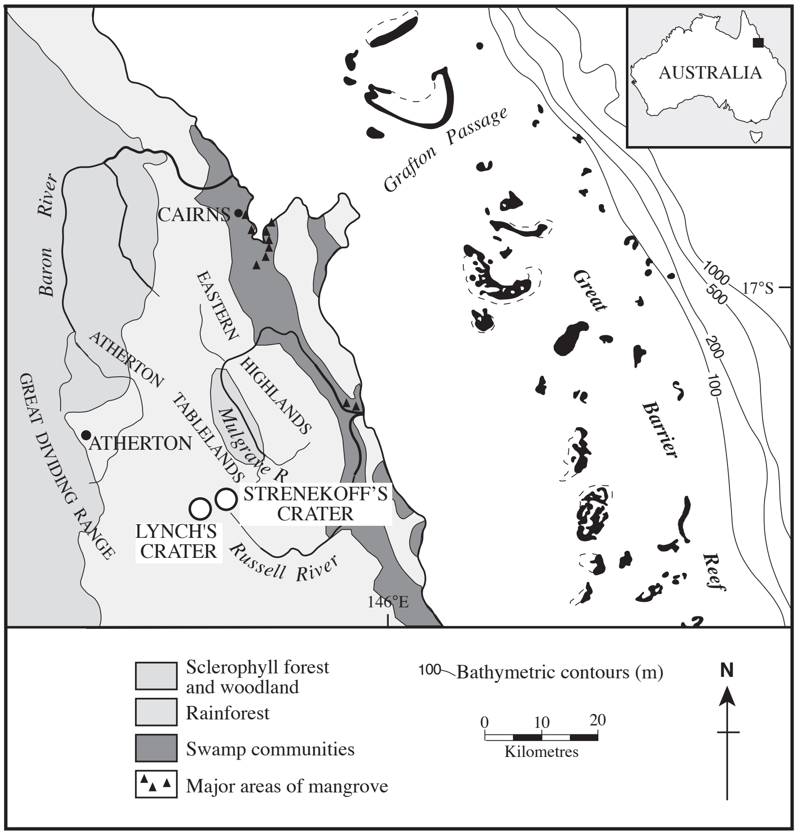

Supplementary Figure 2: Location of Lynch's Crater in northeastern Australia.

Key locations discussed in text.

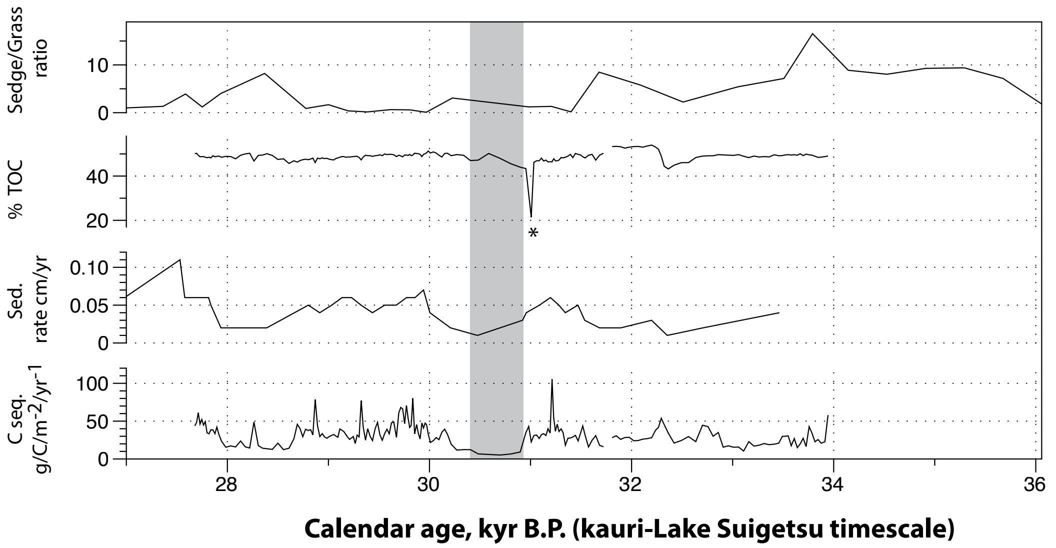

Supplementary Figure 3: Summary environmental changes preserved in Lynch's Crater. Changes in Lynch's Crater carbon flux compared to the grass/sedge ratio, percentage Total Organic Carbon (TOC) and sedimentation rate. Grey column denotes the ~420-year duration downturn in sequestered carbon. The minimum in sediment TOC content previously correlated to HE3 is marked by an asterisk (*)[2].

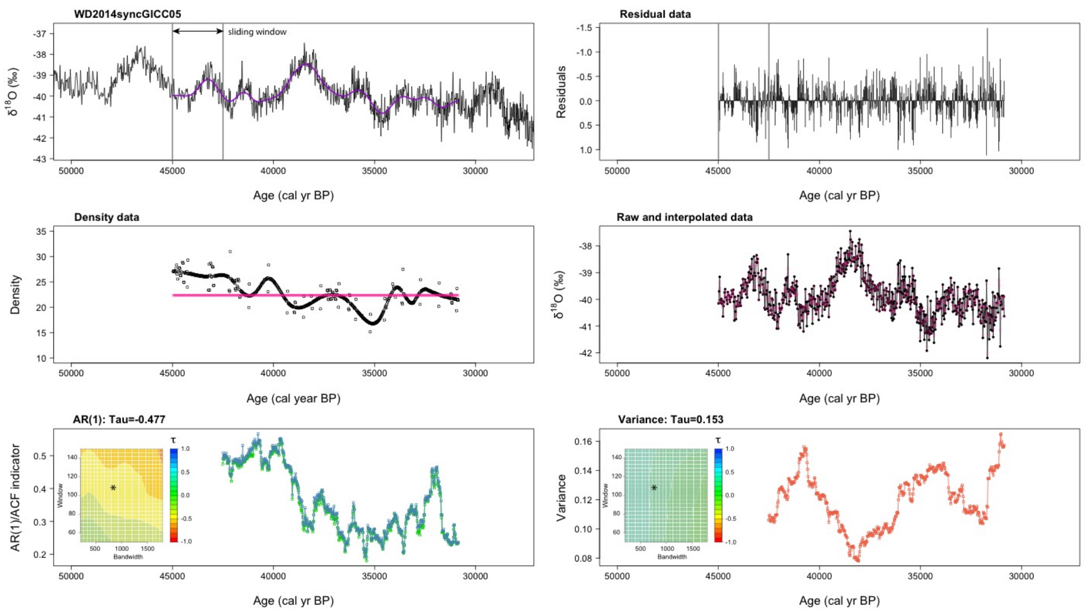

Supplementary Figure 4: Tipping point analysis of the WAIS Divide data.

WD2014$_{sync}$ placed on GICC05 (ref. [3]); residual data after detrending, data density showing how the density of the data points changes over the record; the raw and interpolated data showing how the data were pre-processed, and results of the autocorrelation and variance over a sliding window of 2500 years (as shown between the two grey vertical lines). There is no clear trend in autocorrelation, although there is a slight increase in variance. Included are contour plots showing the Kendall tau values for autocorrelation (left) and variance (right) over a large range of smoothing bandwidths and sliding window sizes for WAIS Divide. The asterisks on the contour plots show the bandwidth and window lengths used for the analysis. These results show that the autocorrelation and variance measured in this dataset are relatively insensitive to these parameter choices.

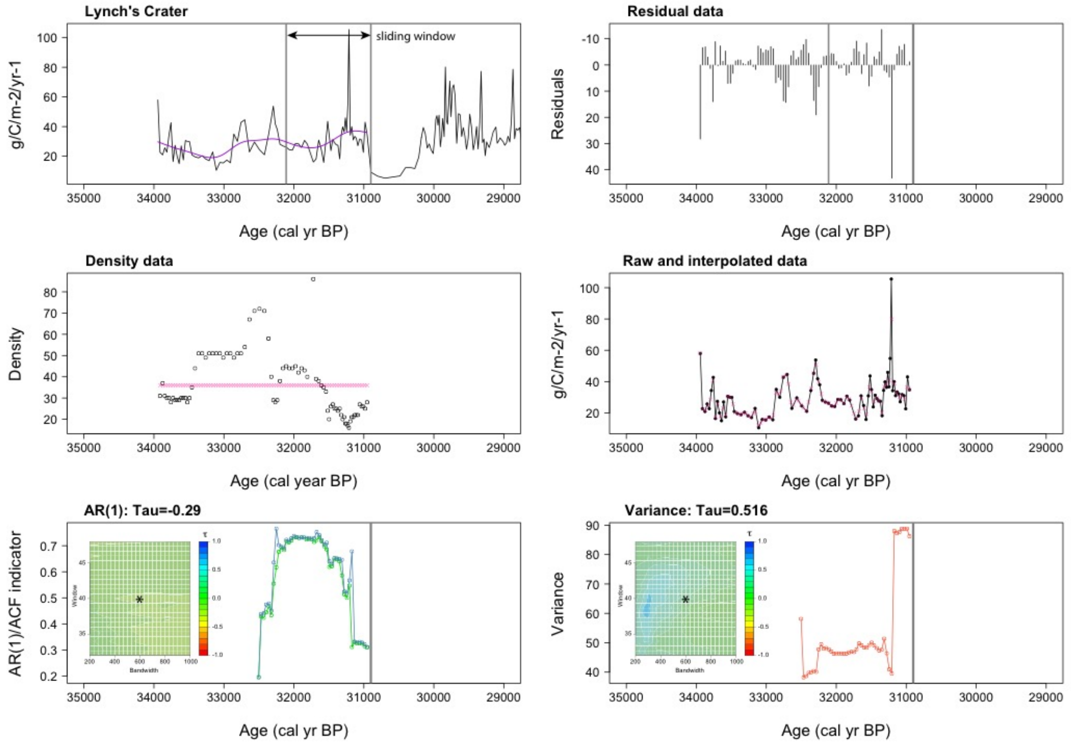

Supplementary Figure 5: Tipping point analysis of the Lynch's Crater carbon accumulation data. Residual data after detrending, data density showing how the density of the data points changes over the record; the raw and interpolated data showing how the data were pre-processed, and results of the autocorrelation and variance over a sliding window of 1100 years (as shown between the two grey vertical lines). There is no clear trend in autocorrelation or variance; although the variance does display one sharp jump near the cut-off point, this appears to be the effect of a single extreme data point, and does not indicate a consistent increase in variance. Included are contour plots showing the Kendall tau values for autocorrelation (left panel) and variance (right panel) over a large range of smoothing bandwidths and sliding window sizes for Lynch's Crater. The asterisks on the contour plots show the bandwidth and window lengths used for the analysis. These results show that the autocorrelation and variance measured in this dataset are relatively insensitive to these parameter choices.

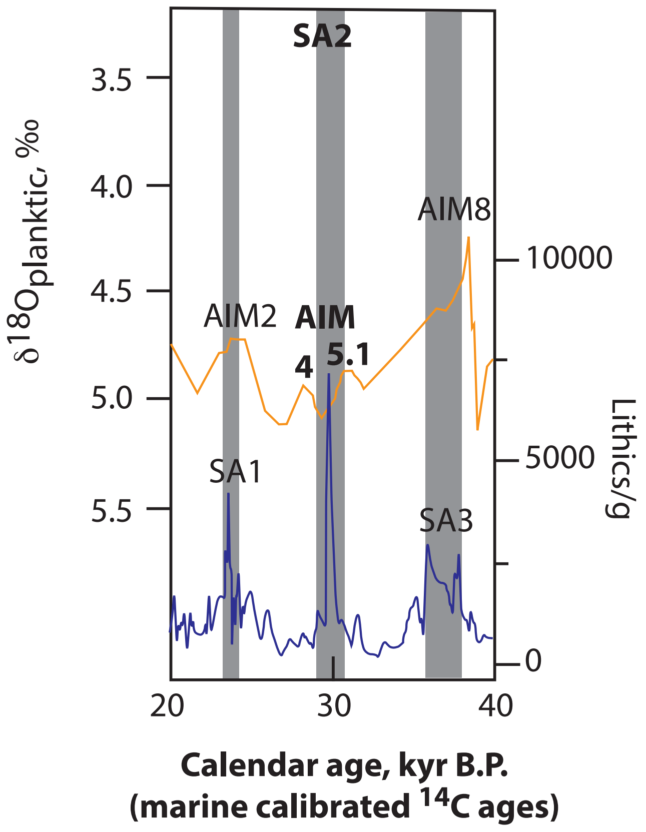

Supplementary Figure 6: Climatostratigraphic position of South Atlantic ice rafted debris layer 2. Relative position of SA2 between planktic $\delta^{18}O$ AIM5.1 and 4 in marine core TTNO57-13/1094 (ref. [4,5]). Chronology developed from ten AMS ^{14}C dates on monospecific samples of *Neogloboquadrina pachyderma* (sin.) to a depth of 9.7 m below seafloor to ~38 kyr BP as reported by Kanfoush *et al*.[5].

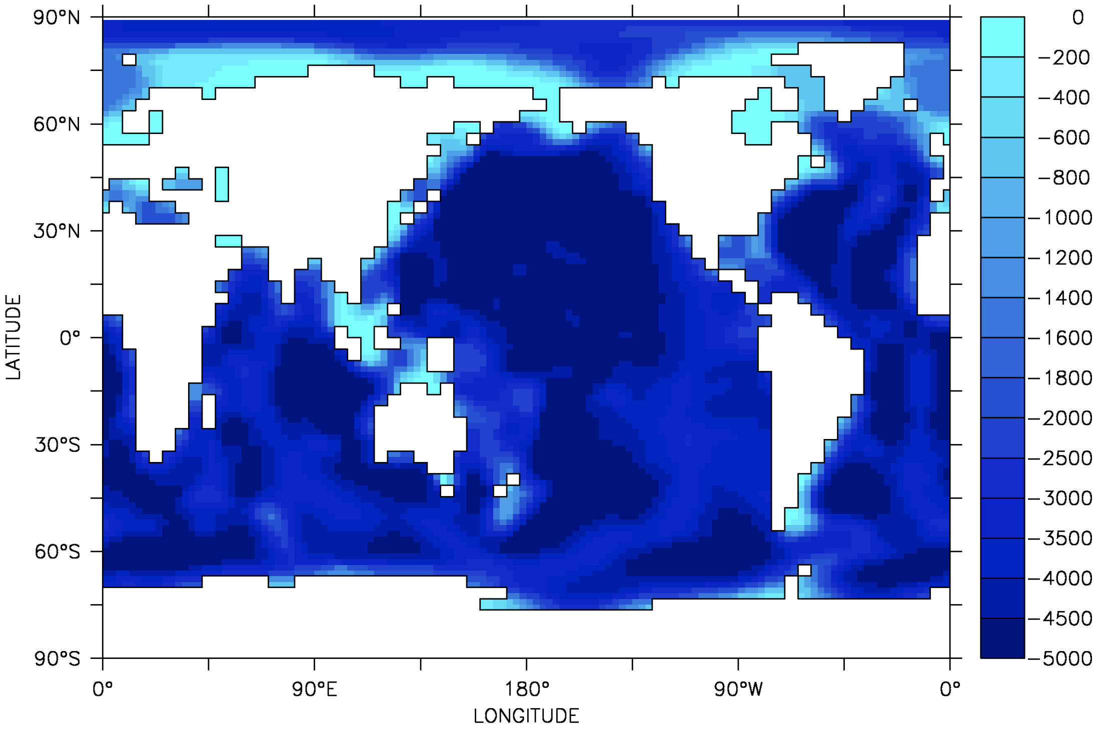

Supplementary Figure 7: Smoothed version of the 2-Minute Gridded Global Relief Data topography. The 2-Minute Gridded Global Relief Data topography (ETOPO2v2) used in the CSIRO Mk3L simulations was obtained from https://www.ngdc.noaa.gov/mgg/global/etopo2.html.

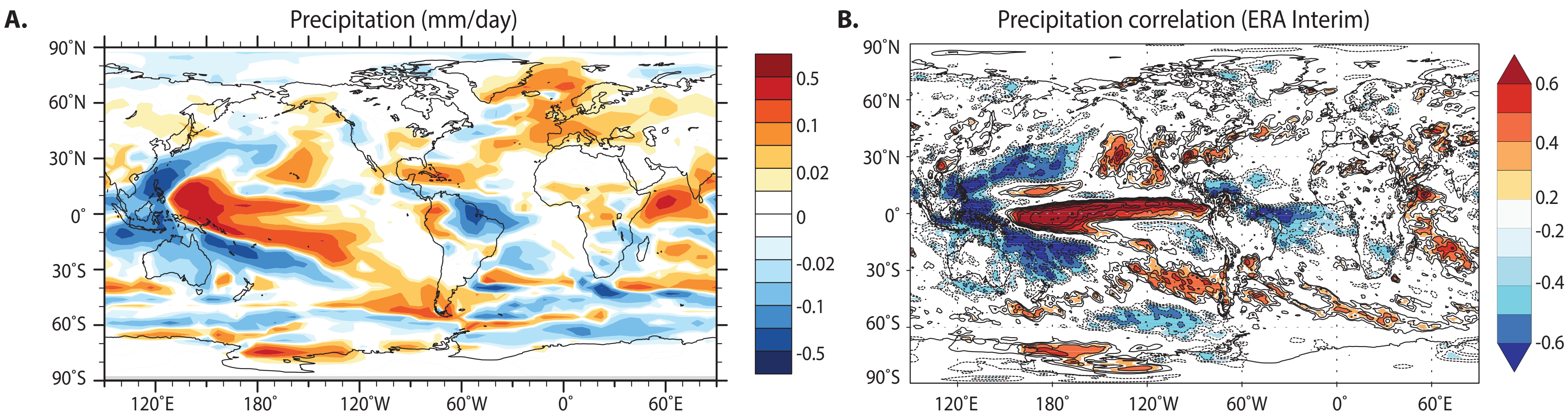

Supplementary Figure 8: Comparison between ensemble mean and contemporary El Niño-Southern Oscillation precipitation changes. Statistically-significant ensemble mean changes in global precipitation (mm/day) (p<0.05; Student t-test) using the CSIRO Mk3L climate system model[6,7] (Panel A.) compared to the relationship between Nino 3.4 SSTs[8] and ERA Interim-derived precipitation[9]. Note the similar spatial relationship across the equatorial Pacific and Atlantic, comparable to other studies investigating the impact of ENSO on global precipitation[10].

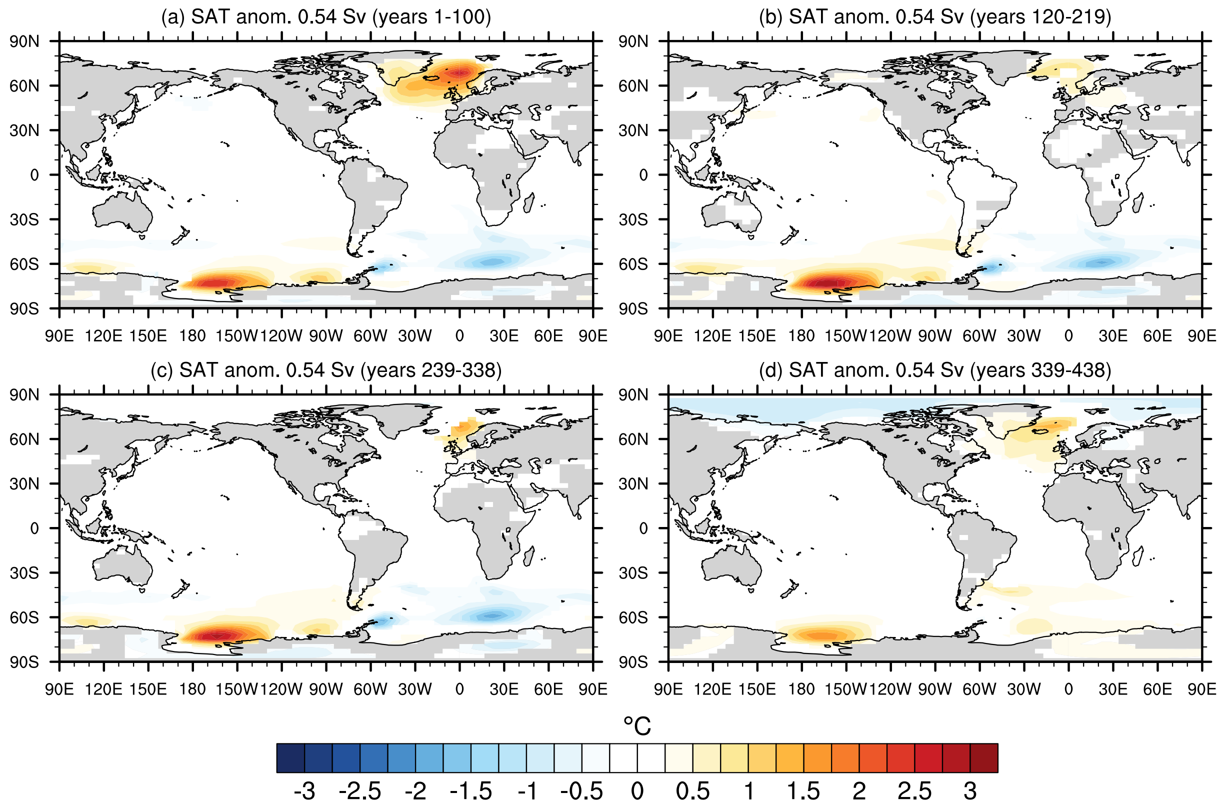

Supplementary Figure 9: Century-long time slices of air temperature anomalies during and after 0.54 Sv freshwater hosing. Statistically-significant ensemble mean 100-year global surface air temperature anomalies using the CSIRO Mk3L climate system model [6,7] during 0.54 Sv freshwater hosing (panels a-c) and the 100 years after the hosing ceases (d). Anomalies are calculated relative to equivalent transient simulations in which no freshwater hosing is applied. Significance $p_{field} < 0.05$.

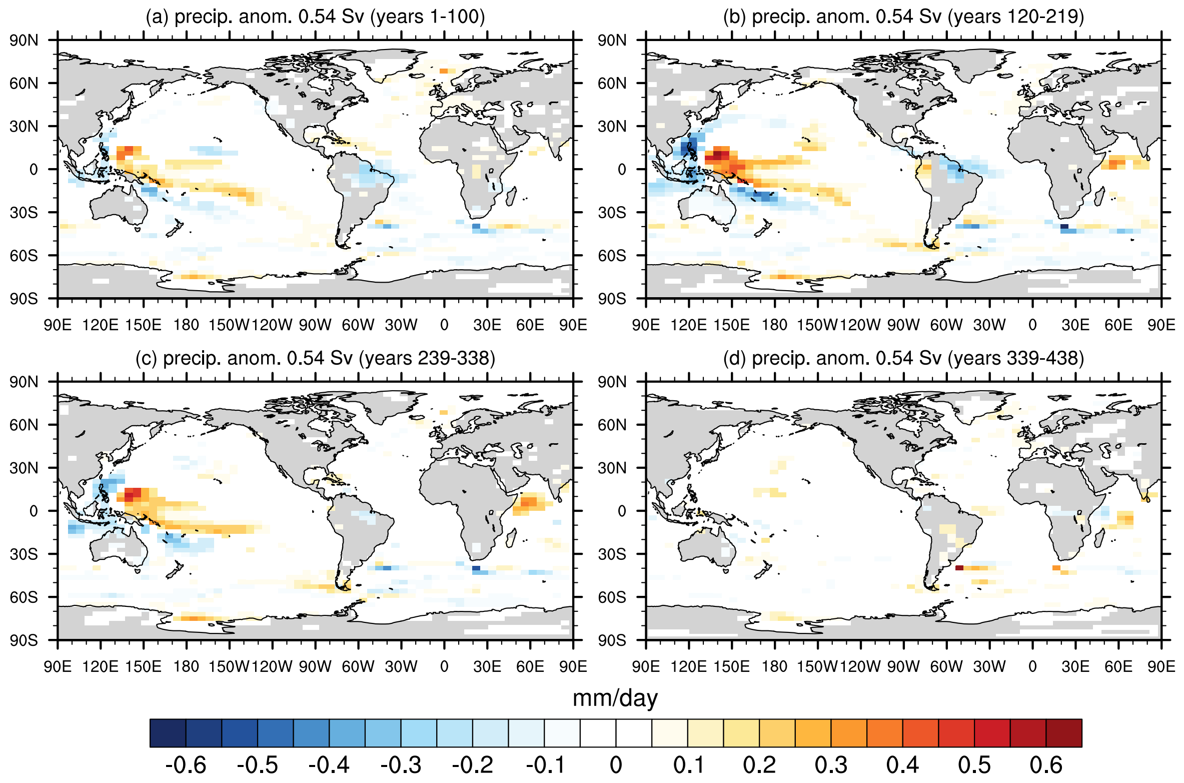

Supplementary Figure 10: Century-long time slices of precipitation anomalies during and after 0.27 Sv freshwater hosing. Statistically-significant ensemble mean 100-year global precipitation anomalies (mm/day) using the CSIRO Mk3L climate system model[6,7] during 0.54 Sv freshwater hosing (panels a-c) and the 100 years after the hosing ceases (d). Anomalies are calculated relative to equivalent transient simulations in which no freshwater hosing is applied. Significance $p_{field} < 0.05$.

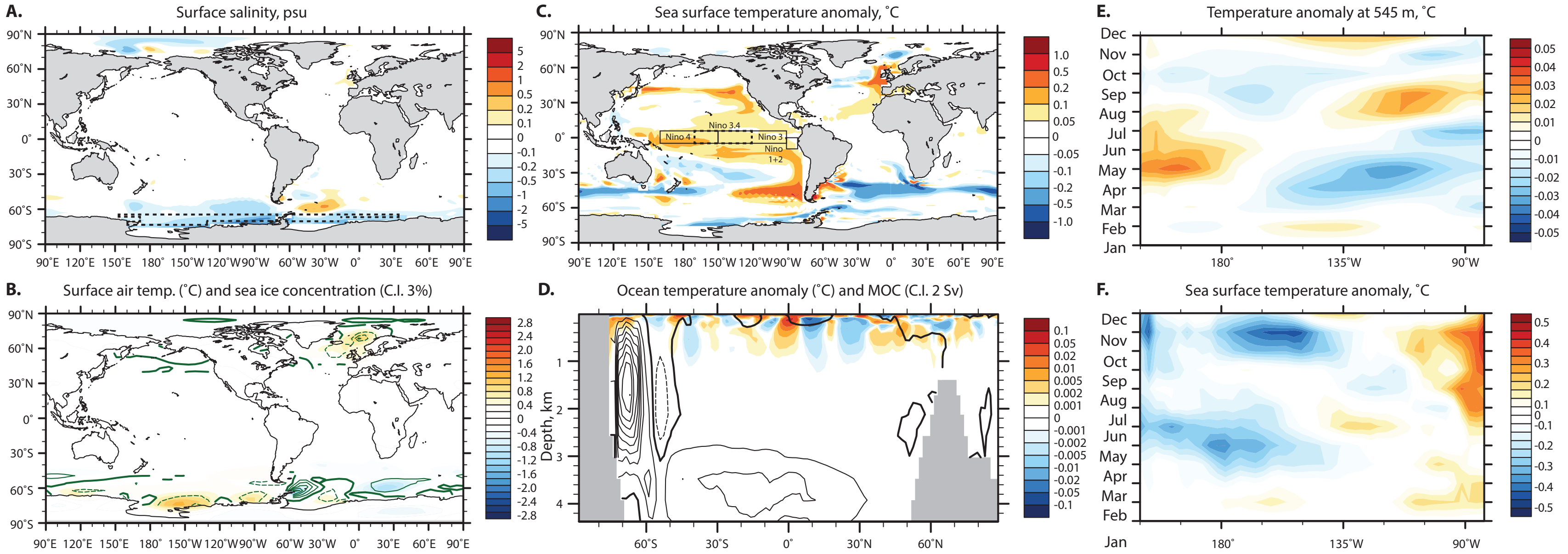

Supplementary Figure 11: Summary of CSIRO Mk3L ensemble simulations showing the impact of a 338-year duration freshwater flux of 0.27 Sv into the Weddell and Ross Seas. Salinity anomaly is shown in Panel A (dashed lines denote regions where freshwater applied with key site locations discussed in text shown). Surface air temperature (colour) and sea ice concentration anomalies (green lines) seen in Panel B are not well correlated with SST anomalies (Panel C), but sea ice concentration increases are highly correlated with salinity decreases in the Ross and Amundsen Sea sectors from the freshwater hosing. Global Meridional Overturning Circulation (MOC) anomaly is shown along with

ocean temperature anomalies in Panel D, where positive contours are solid, negative contours are dashed and the zero contour is emboldened, with a contour interval of 2 Sverdrups (Sv). The Southern positive cell represents reduced Antarctic Bottom Water (AABW) formation. Anomalies in panels A-D are averaged over duration of the 338-year duration freshwater flux. Resulting seasonal equatorial (0°) Pacific eastward propagating Kelvin waves at the thermocline during the first year of freshwater application (545 metres depth) (Panel E) and westward surface propagating Rossby waves (Panel F) identified by temperature changes. Significance $p_{field} < 0.05$.

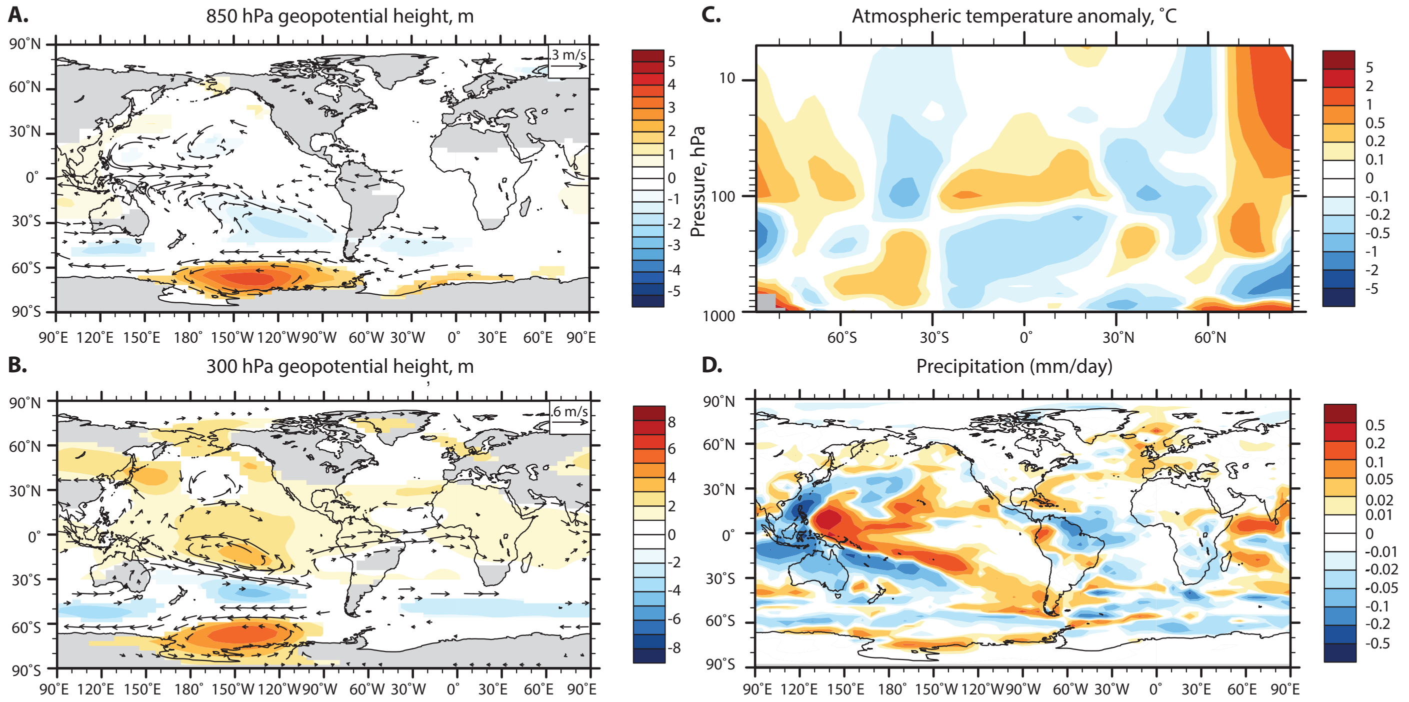

Supplementary Figure 12: Modelled global atmospheric propagation of a 0.27 Sv Southern Ocean freshwater flux during the last glacial period. Geopotential height and wind anomalies at 850 hPa (Panel A) and 300 hPa (Panel B). Zonally averaged global temperature anomalies for the atmosphere (Panel C) reflect the characteristic pattern of westerly propagating Rossby waves. Global annual rainfall anomalies are shown in Panel D. Significance $p_{field} < 0.05$.

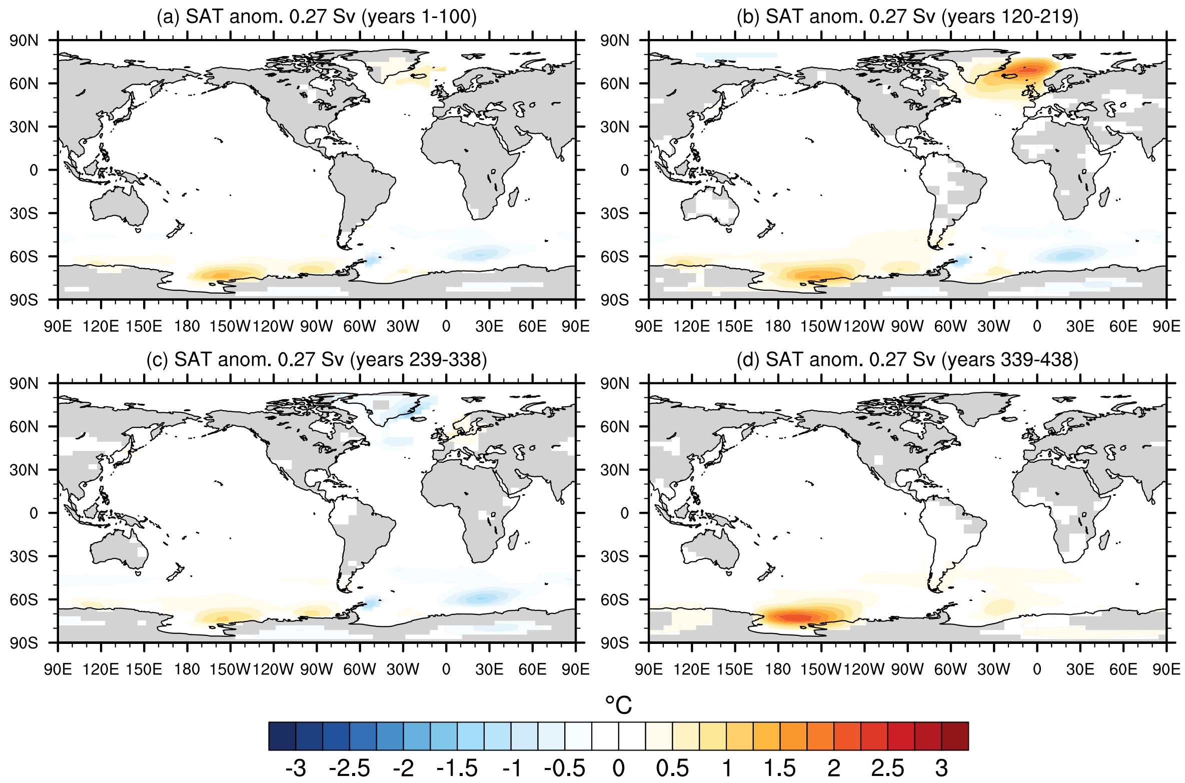

Supplementary Figure 13: Century-long time slices of air temperature anomalies during and after 0.27 Sv freshwater hosing. Statistically-significant ensemble mean 100-year global surface air temperature anomalies using the CSIRO Mk3L climate system model [6,7] during 0.27 Sv freshwater hosing (panels a-c) and the 100 years after the hosing ceases (d). Anomalies are calculated relative to equivalent transient simulations in which no freshwater hosing is applied. Significance $p_{field} < 0.05$.

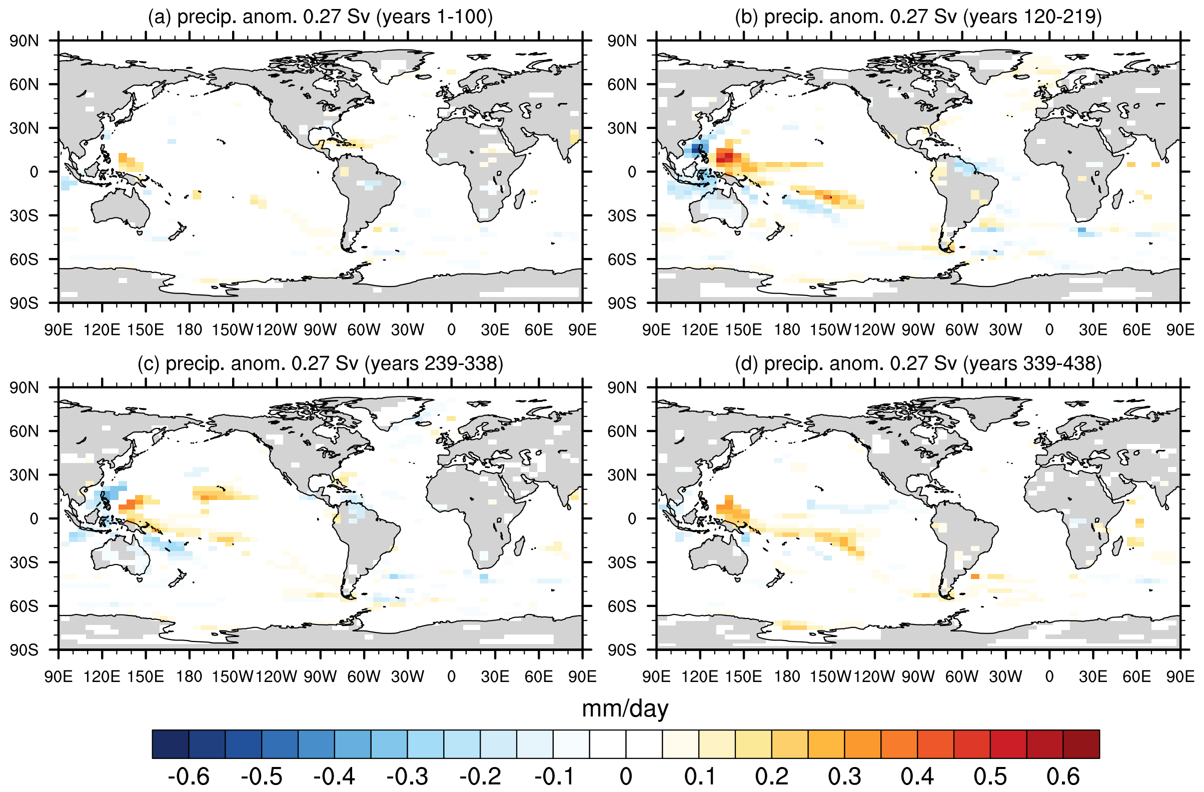

Supplementary Figure 14: Century-long time slices of precipitation anomalies during and after 0.27 Sv freshwater hosing. Statistically-significant ensemble mean 100-year global precipitation anomalies (mm/day) using the CSIRO Mk3L climate system model[6,7] during 0.27 Sv freshwater hosing (panels a-c) and the 100 years after the hosing ceases (d). Anomalies are calculated relative to equivalent transient simulations in which no freshwater hosing is applied. Significance $p_{field} < 0.05$.

Depth, m	^{14}C age	1σ	GICC05 equivalent age BP	1σ	Mean cal age BP	Mean cal 1σ	Comments
0.025					1950	0	Core top
1.315			11653	50	11660	60	Base of Holocene
1.525	10600	100			12340	200	*Globigerina bulloides*
1.815			14642	93	14640	100	Onset of GI-1
2.215	13610	130			15760	180	Combined *Neogloboquadrina pachyderma* (2.215 m)
2.215	13130	80					*Neogloboquadrina pachyderma*
2.395	13370	70			15940	170	*Neogloboquadrina pachyderma*
2.575	14020	70			16810	180	*Neogloboquadrina pachyderma*
2.665	14070	70			16900	160	*Neogloboquadrina pachyderma*
2.755	14070	70			16960	160	*Neogloboquadrina pachyderma*
2.785	14050	90			16990	160	*Neogloboquadrina pachyderma*
2.815	14200	80			17060	170	*Neogloboquadrina pachyderma*
2.905	14510	130			17420	230	*Neogloboquadrina pachyderma*
4.885	20410	220			24370	260	*Neogloboquadrina pachyderma*
5.125	20650	140			24740	240	*Neogloboquadrina pachyderma*
5.725	22200	190			26330	250	*Neogloboquadrina pachyderma*
6.475			28850	449	28580	380	Onset of GI 4
7.225	25430	250			29770	460	*Neogloboquadrina pachyderma*
7.515			31990	554	32110	550	Termination of GI 5.2
7.665			33690	606	33470	460	Onset of GI6
7.785			34690	643	33930	390	Termination of GI 7
8.155			35430	661	34550	390	Onset of GI 7
8.215	30060	380			34610	410	*Neogloboquadrina pachyderma*
8.545			38170	725	37190	580	Onset of GI 8
8.665	32490	490			37430	580	*Neogloboquadrina pachyderma*
9.285			41410	817	41590	730	Onset of GI 10
9.505			43290	868	43170	760	Onset of GI 11
9.955			46810	956	46550	850	Onset of GI 12
10.645			49550	1026	50310	950	Termination of GI 14
10.795			54170	1151	52780	1190	Onset of GI 14

Supplementary Table 1: North Atlantic marine core MD95-2040[1] age model produced using a combination of calibrated radiocarbon ages and inferred Greenland isotope events (on the GICC05 timescale relative to C.E. 1950). See Methods for further details.

Depth, cm	Wk- no	^{14}C age	1σ	Mean cal age BP	Mean cal 1σ	Comments
697.9	36775	29645	181	33874	286	
683.4	36774	29601	187	33463	208	
667.9	36773	28673	162	32707	398	
662.7	36772	27885	149	32353	372	
657.5	36771	27693	146	32198	403	
650.3	36770	27560	151	31893	456	
645.1	32796	27685	294	31682	397	
640.9	32795	27457	275	31536	329	
637.8	32794	27719	283	31469	277	
632.6	32792	27660	281	31344	137	
629.5	32793	27140	279	31281	95	
624.4	32791	27180	273	31197	98	
619.2	32790	26978	258	31088	118	
614	32789	26958	260	30958	160	
613	36769	26812	132	30921	172	
608.8	32788	26070	232	30475	231	
604.7	32394	25809	101	30208	131	
598.4	*32393*	*24359*	*180*			*Significant outlier so excluded*
597.4	32787	25318	212	30004	117	
593.2	32392	25662	213	29941	130	
588	32391	25526	209	29857	138	
582.8	32390	25777	218	29773	142	
577.6	32389	25363	230	29671	147	
572.4	32388	25256	217	29557	158	
567.2	32387	25154	198	29435	159	
562	32386	24881	192	29323	148	
556.8	32385	25258	201	29229	150	
551.6	32384	25246	206	29135	148	
546.4	32383	25059	220	29033	146	
541.1	32786	24855	200	28914	140	
535.9	32785	24868	202	28801	120	
530.7	32784	24771	198	28664	101	Combined with Wk-36768
530.7	36768	24603	109			Combined with Wk-32784
525.5	32783	24223	199	28390	169	
520.3	32782	24196	195	28179	174	
515.1	32781	23638	175	27936	129	
509.9	32780	23820	179	27837	89	
508.5	32767	23783	95	27814	82	
503.5	*32766*	*22506*	*83*			*Significant outlier so excluded*
498.5	*32765*	*17276*	*50*			*Significant outlier so excluded*
494.5	32764	23218	89	27578	59	
489.5	32763	23368	90	27531	59	
465.5	32762	22520	87	26655	269	
405.5	36761	18876	58	22682	241	

Supplementary Table 2: Lynch's Crater radiocarbon ages calibrated against the kauri-Lake Suigetsu dataset used during this study. Those ages with poor agreement index values (<60% and italicized) were excluded prior to the final age model.

**Boundary conditions (range of values)**	**Source with reference(s)**
Orbital parameters	Calculated directly[11]
CO_2 (177-202 ppm)	Taylor Dome/EDML[12,13]
CH_4 (368-426 ppb)	EPICA Dome C (EDC3)[14,15]
N_2O (199-231 ppb)	EPICA Dome C (EDC3)[15,16]

Supplementary Table 3: Sources of greenhouse gas concentrations and orbital parameters used for freshwater hosing experiment reported during this study (32 to 28 kyr BP). Ensembles of experiments were undertaken using the above parameters with 0.27 and 0.54 Sv applied to the Ross and Weddell Seas.

Supplementary References

1 de Abreu, L., Shackleton, N. J., Schönfeld, J., Hall, M. & Chapman, M. Millennial-scale oceanic climate variability off the Western Iberian margin during the last two glacial periods. *Marine Geology* **196**, 1-20 (2003).

2 Muller, J. *et al.* Possible evidence for wet Heinrich phases in tropical NE Australia: the Lynch's Crater deposit. *Quaternary Science Reviews* **27**, 468-475 (2008).

3 WAIS Divide Project Members. Precise interpolar phasing of abrupt climate change during the last ice age. *Nature* **520**, 661-665 (2015).

4 Kanfoush, S. Correlation of ice-rafted detritus in South Atlantic sediments with climate proxies in polar ice over the last glacial period. *The International Journal of Ocean and Climate Systems* **4**, 1-20 (2013).

5 Kanfoush, S. L. *et al.* Millennial-scale instability of the Antarctic Ice Sheet during the last glaciation. *Science* **288**, 1815-1818 (2000).

6 Phipps, S. J. *et al.* The CSIRO Mk3L climate system model version 1.0 – Part 1: Description and evaluation. *Geoscientific Model Development* **4**, 483-509 (2011).

7 Phipps, S. J. *et al.* The CSIRO Mk3L climate system model version 1.0 – Part 2: Response to external forcings. *Geoscientific Model Development* **5**, 649-682 (2012).

8 Rayner, N. A. *et al.* Global analyses of sea surface temperature, sea ice, and night marine air temperature since the late nineteenth century. *Journal of Geophysical Research: Atmospheres* **108**, 4407, doi:4410.1029/2002JD002670 (2003).

9 Dee, D. P. *et al.* The ERA-Interim reanalysis: configuration and performance of the data assimilation system. *Quarterly Journal of the Royal Meteorological Society* **137**, 553-597 (2011).

10 Dai, A. & Wigley, T. M. L. Global patterns of ENSO-induced precipitation. *Geophysical Research Letters* **27**, 1283-1286 (2000).

11 Berger, A. Long-term variations of daily insolation and Quaternary climatic change. *Journal of Atmospheric Sciences* **35**, 2362-2367 (1978).

12 Indermühle, A., Monnin, E., Stauffer, B., Stocker, T. F. & Wahlen, M. Atmospheric CO_2 concentration from 60 to 20 kyr BP from the Taylor Dome Ice Core, Antarctica. *Geophysical Research Letters* **27**, 735-738 (2000).

13 Lüthi, D. *et al.* CO_2 and O_2/N_2 variations in and just below the bubble–clathrate transformation zone of Antarctic ice cores. *Earth and Planetary Science Letters* **297**, 226-233 (2010).

14 Loulergue, L. *et al.* Orbital and millennial-scale features of atmospheric CH_4 over the past 800,000 years. *Nature* **453**, 383-386 (2008).

15 Parrenin, F. *et al.* The EDC3 chronology for the EPICA Dome C ice core. *Clim. Past* **3**, 485-497 (2007).

16 Spahni, R. *et al.* Atmospheric methane and nitrous oxide of the Late Pleistocene from Antarctic ice cores. *Science* **310**, 1317-1321 (2005).
