# Supplementary material for: Rapid global ocean-atmosphere response to Southern Ocean freshening during the last glacial
Source: Nat Commun. 2017 Sep 12;8:520. doi: 10.1038/s41467-017-00577-6 (PMC5595922; doi:10.1038/s41467-017-00577-6)
Supplement: Supplementary file 3 — Supplementary Software 1 [file 41467_2017_577_MOESM3_ESM.rtf]

Supplementary Software 1: OxCal code for modelling radiocarbon ages.Plot(){ Outlier_Model("General",T(5),U(0,4),"t");  Curve("Combined_Suigetsu_Kauri_Calibration_Curve", " Combined_Suigetsu_kauri_Data");  P_Sequence("Lynch’s Crater",1,0.1,U(-2,2))  { Boundary(“7m core depth”);   R_Date("Wk-36775",29645,181)   { Outlier(0.05); z=697.9; };…   R_Date("Wk-36761",18876,58)   { Outlier(0.05); z=405.5;};   Boundary(“4m core depth”);  }; };
